# A musculoskeletal model of human locomotion driven by a low dimensional set of impulsive excitation primitives

**DOI:** 10.3389/fncom.2013.00079

**Published:** 2013-06-26

**Authors:** Massimo Sartori, Leonardo Gizzi, David G. Lloyd, Dario Farina

**Affiliations:** ^1^Department of Neurorehabilitation Engineering, Bernstein Focus Neurotechnology Göttingen, University Medical Center GöttingenGöttingen, Germany; ^2^Pain Clinic, Center for Anesthesiology, Emergency and Intensive Care Medicine University Hospital GöttingenGöttingen, Germany; ^3^Centre for Musculoskeletal Research, Griffith Health Institute, Griffith UniversityGold Coast, QLD, Australia

**Keywords:** EMG-driven modeling, musculoskeletal modeling, lower extremity, multiple degrees of freedom, muscle dynamics, muscle synergy

## Abstract

Human locomotion has been described as being generated by an impulsive (burst-like) excitation of groups of musculotendon units, with timing dependent on the biomechanical goal of the task. Despite this view being supported by many experimental observations on specific locomotion tasks, it is still unknown if the same impulsive controller (i.e., a low-dimensional set of time-delayed excitastion primitives) can be used as input drive for large musculoskeletal models across different human locomotion tasks. For this purpose, we extracted, with non-negative matrix factorization, five non-negative factors from a large sample of muscle electromyograms in two healthy subjects during four motor tasks. These included walking, running, sidestepping, and crossover cutting maneuvers. The extracted non-negative factors were then averaged and parameterized to obtain task-generic Gaussian-shaped impulsive excitation curves or primitives. These were used to drive a subject-specific musculoskeletal model of the human lower extremity. Results showed that the same set of five impulsive excitation primitives could be used to predict the dynamics of 34 musculotendon units and the resulting hip, knee and ankle joint moments (i.e., *NRMSE* = 0.18 ± 0.08, and *R*^2^ = 0.73 ± 0.22 across all tasks and subjects) without substantial loss of accuracy with respect to using experimental electromyograms (i.e., *NRMSE* = 0.16 ± 0.07, and *R*^2^ = 0.78 ± 0.18 across all tasks and subjects). Results support the hypothesis that biomechanically different motor tasks might share similar neuromuscular control strategies. This might have implications in neurorehabilitation technologies such as human-machine interfaces for the torque-driven, proportional control of powered prostheses and orthoses. In this, device control commands (i.e., predicted joint torque) could be derived without direct experimental data but relying on simple parameterized Gaussian-shaped curves, thus decreasing the input drive complexity and the number of needed sensors.

## Introduction

Human movement is the result of the coordinated excitation of musculotendon units (MTUs), which actuate multiple joints in the upper and lower extremities. Because of the inherent redundant nature of the human neuromuscular system, multiple MTU excitation patterns can result in the same joint moment, position, and motion (Tax et al., 1990; Buchanan and Lloyd, 1995). When performing a motor task, the neural drive to MTUs defines the specific excitation patterns among the many possible solutions. Understanding the mechanisms underlying an individual's excitation patterns is an open question in current movement neuroscience and biomechanics. This is fundamental for understanding human locomotion and for the development of novel neurorehabilitation technologies.

The neural drive, or excitation, to MTUs is ultimately determined by action potential trains generated from pools of alpha motor neurons that innervate specific MTUs (Farina and Negro, 2012). Surface electromyography (EMG) indirectly reflects the neural excitation to MTUs and can be easily recorded during human movement. For this reason, EMG signals recorded from the major lower extremity muscle groups have been used to directly drive open-loop forward dynamics simulations using models that are accurate, physiological, and anatomical representations of the human neuromusculoskeletal system (Lloyd and Besier, 2003; Sartori et al., 2012a,c).

It has been shown that the multi-muscular EMG patterns observed during motor behaviors have a lower dimensionality with respect to the number of muscles and associated MTUs (D'Avella et al., 2003; Bizzi et al., 2008). Therefore, the EMG excitation patterns can be expressed using a low-dimensional set of MTU excitation primitives (XPs). In human locomotion, the XPs have been consistently observed to be sequential and minimally overlapped impulses (burst-like) of excitation (Ivanenko et al., 2004, 2006; Cheung et al., 2005; Bizzi et al., 2008). Therefore, human locomotion has been interpreted as being generated by an impulsive excitation of groups of MTUs, with the timing dependent on the biomechanical goal of the task (Ivanenko et al., 2005). The association between XP timing and task performance was also particularly evident in (Oliveira et al., 2013).

Based on this experimental evidence, in this study we propose the use of a low-dimensional set of single-impulse, Gaussian-shaped XPs to drive a physiologically accurate, subject-specific musculoskeletal model of the human lower extremity (Sartori et al., 2012a). Within the musculoskeletal model, the XPs operate as an impulsive controller, where the onset of an XP corresponds to the recruitment of the associated muscles and MTUs.

Although the use of single-impulse, Gaussian-shaped curves was previously supported by experimental evidence from human locomotion studies (Ivanenko et al., 2006), this current study is not focussed on any speculation on the physiological nature of human locomotion excitation patterns. Rather, the use of single-impulse curves in this study has the purpose of exploiting actual primitives of excitations having simple mathematical formalizations. The combination of these single-impulse curves allows generation of more complex multi-impulse excitation inputs and MTU recruitment patterns that might emerge from human locomotion tasks, which have been often observed in the literature (Davis and Vaughan, 1993; Ivanenko et al., 2006; Clark et al., 2010).

Previous studies have used low-dimensional sets of multi-impulse curves within musculoskeletal models of the human lower extremity for the purpose of assessing the mechanical role of muscles during human locomotion (Neptune et al., 2009; McGowan et al., 2010; Allen and Neptune, 2012). Furthermore, other studies assessed and explored the conceptual idea of muscle synergies in relation to the biomechanics of human and animal movement (Zajac et al., 2002; McKay and Ting, 2008; Fregly et al., 2012b; Kutch and Valero-Cuevas, 2012; Ting et al., 2012).

The present study, however, addresses a number of questions that have not been considered in the current literature. One main hypothesis is that a low-dimensional controller of single-impulse XPs could be designed to be generic to subjects and motor tasks, but sufficiently selective to drive a subject-specific musculoskeletal model of the human lower extremity. This would allow coordinating large groups of MTUs and subsequently predicting joint moments about multiple degrees of freedom (DOFs) in the lower extremity during a variety of motor tasks that are substantially different to each other. It is also hypothesized that the use of a *subject-generic, task-generic, low-dimensional* XP set, as opposed to a *subject-specific, task-specific, high-dimensional* EMG set, does not lead to substantial loss of joint moment prediction accuracy in the driven musculoskeletal model. This view would pose the problem of how to differentiate the model's outputs across movements, since the same impulsive controller is used as input drive across different tasks and subjects. In this scenario, it is hypothesized that the model's outputs (i.e., MTU forces and resulting joint moments) are differentiated across movements by the experimental joint kinematics input to the model. This is subsequently used to estimate somatosensory information such as the instantaneous MTU kinematics (Sartori et al., 2012c) and update the MTU force and resulting joint moment estimates (see Methods Section) (Lloyd and Besier, 2003; Sartori et al., 2012a). It is worth noting that the experimental joint kinematics input might, in fact, differ from the kinematics that would be obtained by converting the model's predicted joint moments into joint position. Therefore, within our methodology, the experimental joint kinematics operates as an error correction factor that accounts for somatosensory information (i.e., MTU kinematics) and compensates for the static behavior and simplified structure of the generic XP-based controller. Therefore, prediction discrepancies are not compensated for by a task-specific, subject-specific impulsive controller but rather by joint kinematics information.

Addressing these questions not only would offer further indirect evidence of the theoretical correctness of the human locomotion control scheme previously proposed in the literature (Ivanenko et al., 2005, 2007; Lacquaniti et al., 2012), but would also support the hypothesis that a variety of human locomotion tasks, substantially different from each other, may share a similar neuromuscular control scheme of impulsive nature. Finally, it would provide a novel musculoskeletal model of human locomotion that (1) could be operated in an open-loop forward dynamics way without using numerical optimization to match the experimental joint moments, (2) could be therefore executed at low computational cost (see Results Section), and (3) could produce movement-specific joint moment estimates even if driven by subject-generic and task-generic XPs.

The main advantage of the proposed approach is that, once an XP set has been defined, no EMG recordings are needed for the model operation. This might have substantial implications in the development of novel neurorehabilitation technologies. In this scenario, our proposed XP-driven musculoskeletal model can be operated at low computational costs for the real-time prediction of an individual's neuromusculoskeletal dynamics and for the subsequent development of neuromuscular human-machine interfaces for powered prostheses and orthoses control. In this, MTU activation and joint moments can be predicted in real-time solely from the low-dimensional XP set and three-dimensional joint kinematics without loss of accuracy with respect to using EMG signals. This would substantially decrease the input drive complexity as well as the number of needed sensors on the wearable robot, thus increasing the system robustness.

In this paper, the proposed XP-driven modeling methodology is presented and validated with a direct comparison to the previously presented EMG-driven modeling method (Lloyd and Besier, 2003; Sartori et al., 2012a).

## Materials and methods

The procedure comprised four steps: (1) collecting human movement data using motion capture technology, (2) modeling and simulating the recorded human movement, (3) determining a low-dimensional set of XPs, and (4) calibrating and executing the musculoskeletal model of the human lower extremity.

### Human movement data collection

Two healthy men (age: 28 and 26 years, height: 183 and 167 cm, mass: 67 and 73 kg) volunteered for this investigation and gave their informed, written consent. The project was approved by the Human Research Ethics committee at the University of Western Australia.

The motion data acquired from the two subjects were static anatomical trials, functional calibration trails, and the actual dynamic gait trials. During all trials, the three-dimensional location of retro-reflective markers placed on the subjects' body was recorded (250 Hz) using a 12-camera motion capture system (Vicon, Oxford, UK). During the dynamic trials, ground reaction forces (GRFs) and EMG signals were collected (2000 Hz) synchronously with the marker trajectories using an in-ground force plate (AMTI, Watertown, USA), and bipolar electrodes with a telemetered EMG system (Noraxon, Scottsdale, USA), respectively.

The dynamic trials were eight repetitions of four motor tasks including fast walking (FW, 1.3 ± 0.25 m/s), running (RN, 2.5 ± 0.5 m/s), sidestepping (SS, 2.0 ± 0.35 m/s), and crossover (CO, 1.9 ± 0.15 m/s) cutting maneuvers. Each trial included the full stance phase of gait of the subjects' right leg. The CO and SS tasks were straight running with change of direction to the right and left respectively. In these, the direction change was performed by having the right leg in contact with the floor and going through the full stance phase. In all tasks, velocities were measured by tracking the speed of the trunk markers during the stance phase. The four motor tasks were chosen because (1) they required the production of large joint moments about the six considered DOFs including: hip flexion-extension (HipFE), hip adduction-abduction (HipAA), hip internal-external rotation (HipROT), knee flexion extension (KneeFE), ankle plantar-dorsi flexion (AnkleFE), and ankle subtalar flexion (AnkleSF), and (2) because they reflected different MTU recruitment strategies and contraction dynamics. This permitted us to investigate whether the proposed methodology could use a simple XP set to predict joint moments produced about the six considered DOFs while accounting for different MTU operation strategies.

EMG data were collected from 16 muscle groups including: hip adductors (add), gluteus maximus (gmax), gluteus medius (gmed), gracilis (gra), tensor fasciae latae (tfl), lateral hamstrings (latham), medial hamstrings (medham), sartorius (sar), rectus femoris (recfem), vastus medialis (vasmed), vastus lateralis (vaslat), gastrocnemius medialis (gasmed), gastrocnemius lateralis (gaslat), peroneus group (per), soleus (sol), and tibialis anterior (tibant). Both GRFs and marker trajectories were low-pass filtered with a fourth-order Butterworth filter. Cut-off frequencies (between 2 and 8 Hz) were determined by a trial-specific residual analysis (Winter, 2004). EMGs were processed by band-pass filtering (30–450 Hz), then full-wave rectifying, and low-pass filtering (6 Hz).

From the collected dynamic trials, two distinct datasets were created; one for the calibration of the musculoskeletal model and the other for the validation. For each subject, the calibration dataset included two repeated trials of the four motor tasks (i.e. FW, RN, SS, and CO). A different dataset was used to validate the calibrated musculoskeletal model on each subject and included six repeated novel trials for the four considered motor tasks. None of the trials in the validation dataset were included in the calibration dataset. Therefore, there was no common data between the two datasets.

### Movement modeling

Using the software OpenSim^1^ (Delp et al., 2007), a generic model of the human musculoskeletal geometry^2^ was scaled to match the individual subject's anthropometry. This was done based on the experimentally measured marker positions recorded from the static standing poses, and the location of the hip, knee and ankle joint centers as well as knee flexion-extension axis determined using the functional calibration trials (Besier et al., 2003b). During the scaling process, virtual markers were created and placed on the musculoskeletal geometry model based on the position of the experimental markers. The OpenSim Inverse Kinematics (IK) algorithm (Delp et al., 2007) solved for joint angles that minimized the least-squared error between experimental and virtual markers. The joint moments that needed to track the IK-generated angles were obtained using Inverse Dynamics (ID) and Residual Reduction Analysis (RRA) (Delp et al., 2007). The joint moments produced by this pathway were called “the experimental” moments. The alternative pathway to estimate joint moments was by the XP-driven and EMG-driven models (Sartori et al., 2012a).

**Figure A1:**
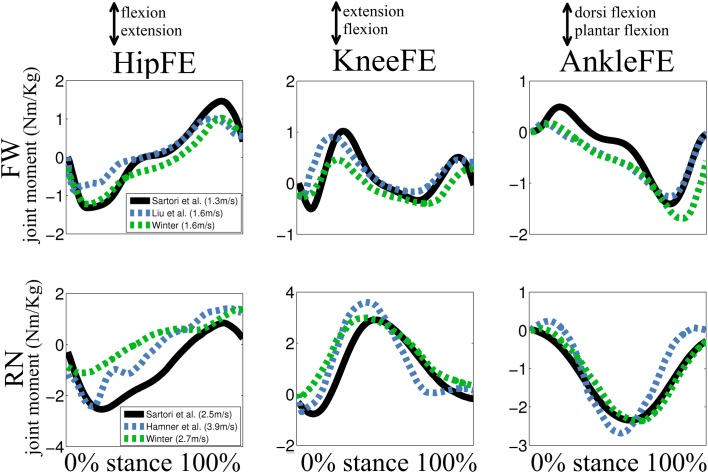
**Experimental joint moments**. Ensemble average curves are depicted for joint moment derived about three degrees of freedom (DOFs) including: hip flexion-extension (HipFE), knee flexion-extension (KneeFE), and ankle plantar-dorsi flexion (AnkleFE) during fast walking (FW) and running (RN). Joint moment derived in our study (Sartori et al., see Methods Section) were compared to data from (Winter, 1983, 2004; Liu et al., 2008; Hamner et al., 2010). The reported data are from the stance phase with 0% being heel-strike and 100% toe-off events.

The estimates produced by our proposed methodology can be directly compared to previously proposed works in the literature (Besier et al., 2009; Winby et al., 2009; Krishnaswamy et al., 2011; Sartori et al., 2012a,b; Hamner and Delp, 2013). Furthermore, Figure A1 compares our derived experimental joint moments to those available in the literature (Winter, 1983, 2004; Liu et al., 2008; Hamner et al., 2010). The normalized root mean squared error and correlation coefficients assumed values that ranged between 0.1–0.3 and 0.89–0.98 respectively (see Validation Procedure Section). During walking, our derived experimental joint moments had peak values of comparable magnitude between the hip extension in the early stance and the ankle plantar flexion in the late stance. This differed to what reported in (Winter, 2004; Liu et al., 2008) in which the peak hip extension moment was smaller than the peak ankle plantar flexion moment. However, it is worth stressing the fact that the use of an ankle joint with oblique axes, like that used in our proposed work, directly coupled ankle plantar-dorsi flexion with ankle subtalar flexion (Kirby, 2001; Yamaguchi et al., 2009). The effect of this coupling on the ankle plantar-dorsi flexion moments is less pronounced in ankle joint models with orthogonal axes (Winter, 2004) or in models in which the ankle subtalar flexion is constrained to the anatomical neutral position (Liu et al., 2008).

### Muscle excitation primitive

To identify the muscle XPs, a two-step process was used based on a previously described non-negative matrix factorization (NNMF) technique (Lee and Seung, 2001). First, the muscle-specific EMG linear envelopes were normalized in time and with respect to the peak processed EMG values obtained from all trials (Ivanenko et al., 2004; Gizzi et al., 2011). In this way, each muscle for each trial and motor task was equally represented in the final muscle weighting computation and results reflected only changes in timing. The normalized linear envelopes computed from all dynamic trials in the validation dataset collected from the two subjects were then combined into an *m* × *n* matrix, where *m* indicates the number of muscles and *n* the number of trial frames × number of trials × number of subjects. That is, each row was associated to a muscle, which concatenated the muscle's EMG data from all trials and subjects. The NNMF was then applied to the *m* × *n* matrix with a number of non-negative factors identified together with their associated weightings (see Results Section). The extracted, experimental non-negative factors were linearly combined with their associated weightings to produce an *m* × *n* matrix of reconstructed EMGs and then compared to the original EMG matrix. The agreement between the two matrices was then quantified by least squares errors. The NNMF was then iterated within an optimization procedure by adjusting the non-negative factors until they minimized the least squared error between experimental and reconstructed EMG data. In this procedure the dimensionality of the non-negative factor set was increased until the accuracy of the reconstructed EMG data exceeded a pre-defined threshold. This was assessed by means of the Variation Accounted For (VAF) index, which was defined as VAF = 1 – SSE/TSS, where SSE (sum of squared errors) represented the unexplained variation and TSS (total sum of squares) was the total variation of the EMG data. A minimal VAF value of 80% was the threshold to be exceeded in this study to consider the reconstruction quality as satisfactory (Gizzi et al., 2011). This resulted in a matrix of five non-negative factors (i.e. 5 × *n* non-negative factor matrix) that accounted for 89% of the variability in the EMG data. In this, each muscle group had five associated weighting factors. These determined how much each of the five non-negative factors contributed to the excitation of a specific muscle group. The weightings were then normalized to the highest weighting value:${\bar{W}}_{j,i}=\frac{W_{j,i}}{\max(W_{1,i},W_{2,i},W_{3,i},W_{4,i},W_{5,i})}$, where *W*_*j,i*_ represented the *j*^th^ weighting (1 ≤ *j* ≤ 5) for the i^th^ muscle group (1 ≤ *i* ≤ 16), whereas ${\bar{W}}_{j,i}$ was the resulting normalized weighting (i.e., 0 ≤ ${\bar{W}}_{j,i}$ ≤ 1).

In the second step, from the 5 × *n* non-negative factor matrix, each non-negative factor that was associated to a specific trial and subject was isolated based on the frames associated to the specific trial. This allowed removing the discontinuities existing between two adjacent non-negative factors. Then, the extracted trial-specific non-negative factors were averaged across trials. The five trial-averaged non-negative factors were then fitted with five Gaussian-shaped single-impulse curves (see Results Section), which represented the XPs that were used to drive the proposed XP-driven musculoskeletal model:

(1) $$g(t)=h\cdot e^{-\frac{{\left(t-b\right)}^{2}}{2\cdot c^{2}}}-s$$

where *t* is the time frame and *h*, *b*, *c*, and *s* were the function parameters defining the Gaussian curve peak height, the position of the center of the peak, the width of the curve bell, and the vertical shift respectively. The function parameters were identified using a simulated annealing optimization algorithm (Goffe et al., 1994) that minimized the root mean squared error with respect to each of the five average non-negative factors.

### Musculoskeletal modeling

**Figure 1:**
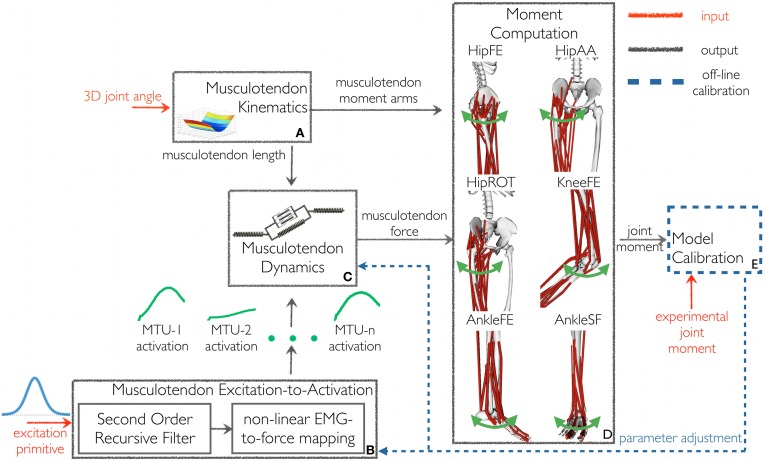
**The schematic structure of the excitation primitive (XP)-driven musculoskeletal model**. It comprises five components: **(A)** Musculotendon Kinematics, **(B)** Musculotendon Excitation-to-Activation, **(C)** Musculotendon Dynamics, **(D)** Moment Computation, and **(E)** Model Calibration. The XP-driven model is initially calibrated using the Model Calibration component. After calibration the model is operated in open-loop. The Musculotendon Excitation-to-Activation component is used to map the initial five-dimensional XP set to the 34 individual MTU activations. Subsequently, MTU force and the resulting moments are determined as a function of MTU activation and experimental three-dimensional musculotendon kinematics (i.e., calculated using Inverse Kinematics), without tracking experimental joint moments. Joint moments are predicted with respect to six degrees of freedom: hip flexion-extension (HipFE), hip adduction-abduction (HipAA), hip internal-external rotation (HipROT), knee flexion-extension (KneeFE), ankle plantar-dorsi flexion (AnkleFE), and ankle subtalar-flexion (AnkleSF).

The XP-driven musculoskeletal model (Figure 1) was developed from our previously described EMG-driven model of the human lower extremity (Lloyd and Besier, 2003; Winby et al., 2009; Sartori et al., 2012a,c). The following of this section provides a description of the XP-driven modeling workflow as well as a description of the model components.

**Figure 2:**
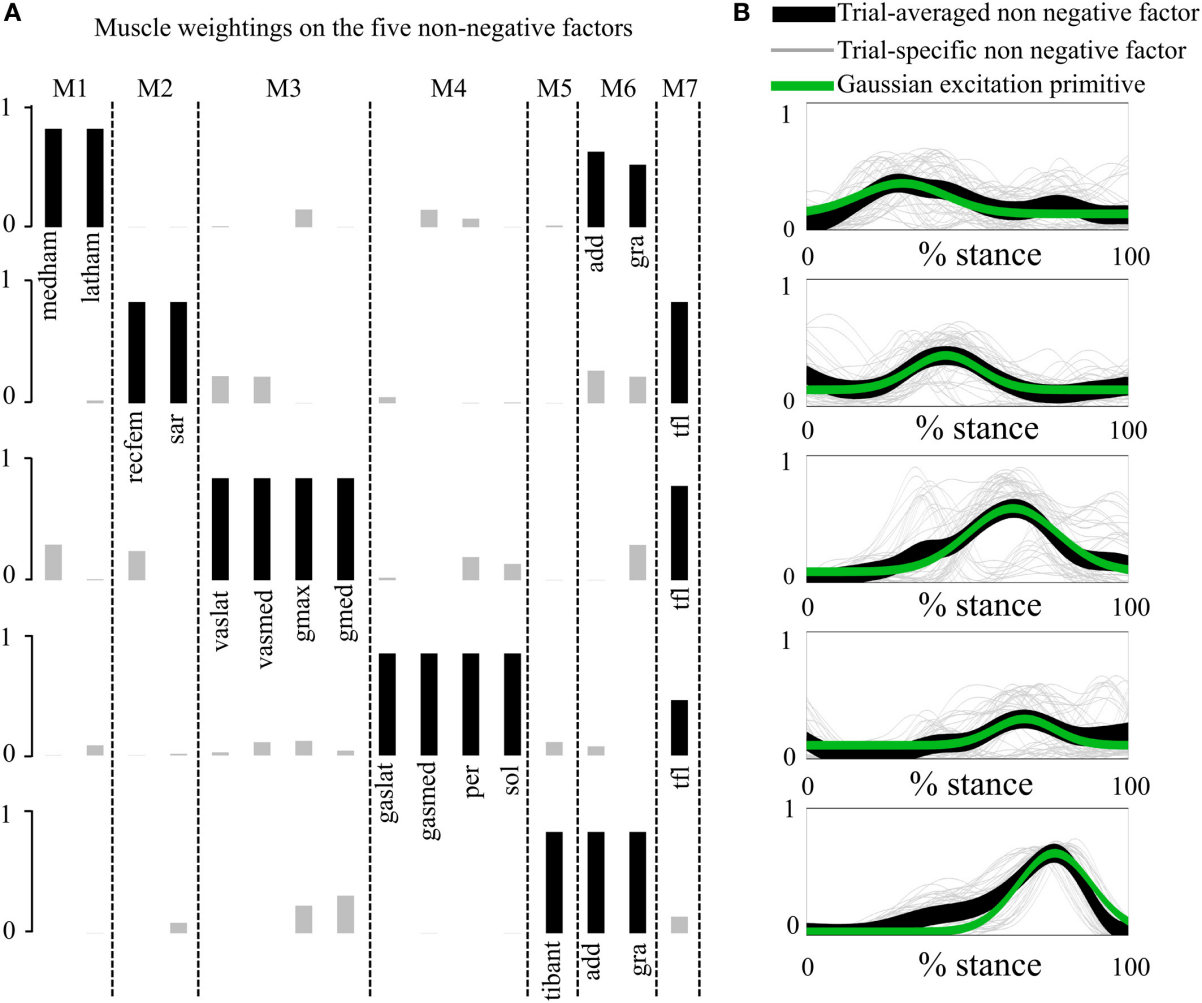
**Non-negative factors, muscle weightings, muscle modules, and associated parameterized Gaussian excitation primitives (XPs). (A)** Muscle modules (i.e., M1–M7) show how the weightings (i.e., black and gray bars) of the XPs contribute to the excitation of groups of muscles. Black bars show the weightings with a value greater than 0.4. **(B)** The five non-negative factors extracted from each motor trial (trial-specific non-negative factors) are averaged across trials (trial-averaged non-negative factors), which enabled parameterized Gaussian excitation primitives (XPs) to be created. The reported data are from the stance phase with 0% being heel-strike and 100% toe-off events.

In the proposed *XP-driven modeling workflow*, a five-dimensional impulsive controller (i.e. made of five XPs, Figure 2) defined, a priori, an initial recruitment scheme for 34 MTUs in the human lower extremity. The properties of the recruitment scheme were preserved across subjects and motor tasks including the relative position and peak amplitude of one XP with respect to another and the MTUs recruited by each XP. The five XPs were only time-scaled (i.e. stretched or compressed) to match the length of the stance phase across movements. A closed-loop calibration step (Figure 1E, also see below in this section) was then performed to identify a number of musculoskeletal model parameters, which varied non-linearly across subjects because of anatomical and physiological differences (Sartori et al., 2012a). In this step, the impulsive controller was further refined to determine a finer mapping between the low-dimensional set of XPs and the high-dimensional set of MTU-specific activations (Figure 1B) that best described the MTU-specific activation strategies across the four selected tasks.

The calibrated XP-driven model was then validated on the same motor tasks selected for calibration (i.e. FW, RN, SS, and CO) but using a novel set of trials (see the Human movement data collection Section). During the validation step, the calibrated XP-driven model operated as an open-loop predictive system, which did not use numerical optimization to track the experimental joint moments, and therefore operated at low computational cost (see Results Section). The MTU-specific activations and the resulting joint moments were directly determined as a function of the five XPs and the three-dimensional joint kinematics, i.e. there was no need to record further EMG data.

The proposed model's structure comprised five main components (Figure 1): Musculotendon Kinematics, Musculotendon Excitation-to-Activation, Musculotendon Dynamics, Moment Computation, and Model Calibration.

The *Musculotendon Kinematics component* (Figure 1A) used MTU-specific multidimensional spline functions to produce instantaneous estimates of MTU length ℓ^*mt*^, and three-dimensional moment arms *r* as a function of joint angles (Sartori et al., 2012c)^3^.

The *Musculotendon Excitation-to-Activation component* (Figure 1B) mapped the five XPs into the 34 MTU-specific activations. The five XPs were initially associated to the 16 muscle groups from which the EMG data were recorded. In this, if a muscle group had an associated weighting factor greater than 0.4 on one of the five XPs, then the muscle group was considered to be associated to that specific XP that defined its initial excitation (Neptune et al., 2009). With respect to (Neptune et al., 2009), our proposed cut-off criterion differed in the fact that weightings and primitives were extracted from the matrix concatenating all EMG linear envelopes from all subjects and trials (see Muscle Excitation Primitive Section). Therefore, NNMF generated a single set of weightings that applied to all subjects' trials. In (Neptune et al., 2009), NNMF was individually applied to each subject. This created subject-specific weightings, which were then averaged prior to the application of the 0.4 cut-off criterion. In our proposed methodology, if a muscle group had more than one associated XP, then the average across the XPs was calculated and used as the muscle group initial excitation. Muscle weightings allowed arranging muscles groups into seven modules (see second test results in Results Section). A module included all muscle groups with a weighting greater than or equal to 0.4 on the same XP. All MTUs from all muscle groups within a module received the same initial XP. The XPs were also assigned to MTUs for which experimental EMG data could not be recorded and included the gluteus minimus, iliacus, psoas, and vastus intermedius. In this allocation, two MTUs that shared the same innervation and contributed to the same mechanical action were assumed to be in the same module and have therefore the same initial XP (Kahle and Frotscher, 2002; Ivanenko et al., 2006). Therefore, the XP that was assigned to both the rectus femoris and the sartorius (module 2 in Figure 2A) was also assigned to the iliacus and psoas. The vastus medialis and vastus lateralis XP (module 3 in Figure 2A) was assigned to the vastus intermedius, and the gluteus medius XP to the gluteus minimus (also module 3 in Figure 2A). These assignments were motivated by anatomical and functional information on the MTUs, assuming that the dimensionality computed from a smaller set of MTUs could be applied to the entire set. Furthermore, if the XPs were the reflection of the spinal circuitry dynamics then the XPs must apply to all lower extremity MTUs whether or not experimental EMGs were available for a specific MTU. This mapping enabled us to use the low dimensional set of XPs to excite a larger number of MTUs than those for which experimental EMG data were available. It is worth noting that this mapping is, in general, not entirely described by the muscle group weightings extracted from the available experimental EMG data using NNMF. This is because experimental EMGs do not directly reflect the activity of deeply located MTUs. For this reason, muscle group weightings were not used to linearly combine XPs together. This allowed us to account for the impulsive nature of the MTU recruitment. The transformation from the XP (i.e. applied to a group of MTUs) to the MTU-specific activation (i.e., applied to a single MTU only) is discussed below.

Each XP that was assigned to a group of MTUs was processed by a critically damped second order recursive filter, which simulated the individual MTU twitch response to the initial XP excitation (Thelen et al., 1994; Lloyd and Besier, 2003):

(2) $$u(t)=\alpha \cdot x(t-d)-\beta_{1}u(t-1)-\beta_{2}u(t-2)$$

where *x(t)* was the XP at time *t*, *u(t)* was the MTU-specific post-processed XP, α was the MTU-specific gain coefficient, and β_1_, and β_2_ were the MTU-specific recursive filtering coefficients. The term *d* was the electromechanical delay. This was set to 10ms based on previously reported experimental results (Nordez et al., 2009) and it was treated as a global parameter as previously suggested (Heine et al., 2003).

The resulting *u(t)* signal was then further processed using the non-linear transfer function in Equation 3 (Lloyd and Besier, 2003; Buchanan et al., 2004). This accounted for the non-linearity between the MTU excitation and force, reflecting the saturation at high levels of the motor unit recruitment in generating force (Lloyd and Besier, 2003; Buchanan et al., 2004; Farina and Negro, 2012):

(3) $$a(t)=\frac{e^{Au(t)}-1}{e^{A}-1}$$

where *A* was the non-linear shape factor, which was constrained to − 3 < A < 0, with 0 being a linear relationship (Lloyd and Besier, 2003). The resulting MTU-specific activation, *a(t)*, represented the ultimate control input to the MTU contractile component. Note that this transformation adjusted the timing (Equation 2) and shape (Equations 2 and 3) of the XPs for each MTU individually. This is important to account for the different activation timing emerging from different motor tasks (Ivanenko et al., 2005).

In the *EMG-driven model*, the EMG linear envelopes were directly used to drive the musculoskeletal model. As previously described, EMG linear envelopes were normalized with respect to the peak processed EMG values obtained from the entire set of recorded trials (Sartori et al., 2012a,b). In this scenario, a dedicated EMG linear envelope was associated with each muscle group individually, with all MTUs within a muscle group receiving the same EMG pattern (Sartori et al., 2012a). This accounted for the different excitation dynamics across muscle groups as opposed to when using XPs, which excited multiple muscle groups simultaneously. Therefore, the excitation-to-activation transformation (Equations 2 and 3) could be treated as a global transformation that applied equally to all MTUs. That is, the same values for the filtering coefficients and the shape factor were used for all MTUs in the model. In this context, the deeply located iliacus and psoas MTUs were not driven by EMG signals. As a result, only their passive force contribution was modeled using the Musculotendon Dynamics component (Figure 1C, see below) by setting the MTU activation to zero (Sartori et al., 2012a).

In the *Musculotendon Dynamics component* (Figure 1C), each MTU was modeled as a Hill-type muscle model. In this, the muscle fibers had generic force-velocity *f*(*v*^*m*^), force-length passive *f*_*P*_ (*l*^*m*^), and active *f*_*A*_(*l*^*m*^) curves, which were normalized to maximum isometric muscle force (*F*^max^), optimal fiber length, and maximum muscle contraction velocity (Zajac, 1989). The tendon dynamics was modeled using a non-linear force-strain function *f*(ε) normalized to *F*^max^ (Zajac, 1989). Using biomechanical parameters from (Delp et al., 1990; Lloyd and Buchanan, 1996, 2001), the MTU force *F*^*mt*^ was calculated as a function of *a*(*t*), fiber length *l*^*m*^ and fiber contraction velocity *v*^*m*^:

(4) $$\begin{array}{l} F^{mt}=F^{t}=F^{m}\cos(\phi (t))\\ \;=\left[a(t)f_{A}(l^{m})f(v^{m})+f_{P}(l^{m})\right]F^{\max}\cos(\phi (t)) \end{array}$$

where *F*^*t*^ and *F*^*m*^ were the tendon and fiber force, and ϕ(*t*) the pennation angle. During the process of MTU force estimation, *l*^*m*^ and *v*^*m*^ were determined at each time point while ensuring equilibrium between *F*^*t*^ and *F*^*m*^ in Equation 4 (Lloyd and Besier, 2003).

The Moment Computation component (Figure 1D) estimated the joint moments *M*_*X*_ as the sum of the product of *r*_*X*_ and *F*^*mt*^, for each *X* DOF, i.e.,HipFE, HipAA, HipROT, KneeFE, AnkleFE, and AnkleSF.

The *Model Calibration component* (Figure 1E) determined the values for a set of parameters that vary non-linearly across subjects and cannot be determined experimentally or from literature (Winby et al., 2008). Parameters were varied within predefined boundaries to ensure MTUs always operated within their physiological range (Lloyd and Besier, 2003). Parameters were adjusted using a simulated annealing algorithm (Goffe et al., 1994) until the objective function *f*_*E*_ = (*E*_HipFE_ + *E*_HipAA_ + *E*_HipROT_ + *E*_KneeFE_ + *E*_AnkleFE_ + *E*_AnkleSF_) was minimized equally for each DOF. Each DOF error term (*E*_HipFE_, *E*_HipAA_, *E*_HipROT_, *E*_KneeFE_, *E*_AnkleFE_, *E*_AnkleSF_) was the sum of the root mean square differences between the predicted and experimental joint moments calculated over the eight calibration trials recorded for a specific subject.

During calibration, two MTU-specific activation-filtering coefficients in the Musculotendon Excitation-to-Activation component (Figure 1B) were adjusted, while being constrained to realize a stable positive solution and a critically damped impulsive response for the recursive filter (Equation 2) (Lloyd and Besier, 2003). In this, the two adjusted parameters determined the final value of α, β_1_, and β_2_ in Equation 2. The MTU-specific global shape factor parameter *A* (Equation 3) was also altered between −3 and 0 to account for the non-linear EMG-to-force relationship (Lloyd and Besier, 2003; Buchanan et al., 2004; Winby et al., 2009).

In the Musculotendon Dynamics component, 11 muscle strength coefficients were calibrated to scale the MTU-specific *F*^max^ to match the person's strength, while maintaining the force generating capacity across MTUs. Strength coefficients were varied between 0.5 to 2 and gathered MTUs in 11 groups according to their functional action including uniarticular hip flexors, uniarticular hip extensors, hip adductors, hip abductors, uniarticular knee flexors, uniarticular knee extensors, uniarticular ankle plantar flexors, uniarticular ankle dorsi flexors, biarticular quadriceps, biarticular hamstrings, and biarticular calf muscles. Muscle tendon slack length *l*^*t*^_*s*_, and optimal fiber length *l*^*m*^_*O*_ were also adjusted so that *l*^*t*^_*s*_ =initial value± 5% and *l*^*m*^_*O*_ =initial value± 2.5% with initial values obtained using the methodology presented in (Winby et al., 2008).

## Validation procedure

The validation comprised four tests to assess the XP-driven model prediction ability and to compare it to the EMG-driven model prediction ability. Furthermore, one additional test was performed to assess the XP-driven model computation time.

In the four prediction tests, the subject-specific calibrated XP-driven and EMG-driven models were operated in open-loop, (i.e. without using optimization to track the experimentally recorded joint moments) on each individual motor trial performed by each subject. In this, both the XP-driven and EMG-driven models predicted *a(t)*, and *M*_*X*_ solely using the parameterized XPs, or experimental EMGs respectively, and the three-dimensional joint angles. The models' outputs were then time-normalized using a cubic spline and the similarity between the predicted and the experimental variables was quantified using the coefficient of determination *R*^2^ (i.e., square of the Pearson product moment correlation coefficient) and the normalized root mean squared deviation *NRMSD*:

(5) $$NRMSD=\frac{\sqrt{\frac{1}{N}\sum_{i=1}^{N}{\left({\hat{X}}_{i}-X_{i}\right)}^{2}}}{\max(\hat{X},X)-\min(\hat{X},X)}$$

where *X* and $\hat{X}$ referred to the two variables being compared, which were (1) the predicted and experimental joint moments, (2) the *a(t)* produced using the EMG-driven model and the XP-driven model, or (3) the experimental and parameterized XP curves. Furthermore, *N* referred to the number of points in the considered curves. In our proposed study, these metrics identified acceptable results for values of *NRMSD* and *R*^2^ being 0.0 ≤ NMRSD ≤ 0.3 and 0.7 ≤ *R*^2^ ≤ 1.0. This criterion was based on the results previously proposed in the literature that used EMG-driven methodologies (Besier et al., 2003a; Lloyd and Besier, 2003; Buchanan et al., 2004, 2005; Winby et al., 2009; Manal et al., 2011; Shao et al., 2011).

For the only purpose of displaying results in a concise way, in some cases, the time-normalized models outputs from the same motor task were averaged across trials and/or across subjects. This produced ensemble average curves for the predicted *a(t)*, and *M*_*X*_ as well as for the matching experimental joint moments ${\hat{M}}_{X}$.

The *first test* examined the five non-negative factors that were extracted from the experimental EMG data as well as the five XPs that were parameterized using Equation 1. The agreement between the *j*^th^ experimental non-negative factor (*g*^*j*^_exp_, 1 ≤ *j* ≤ 5) and the corresponding *j*^th^ parameterized XP curve (*g*^*j*^_par_, 1 ≤ *j* ≤ 5) was then quantified using the *R*^2^ and the *NRMSD* coefficients. Furthermore, for the *i*^th^ muscle group (1 ≤ *i* ≤ 16), the value of the five associated normalized weightings ${\bar{W}}_{j,i}$ (1 ≤ *j* ≤ 5) was analyzed. If ${\bar{W}}_{j,i}$ was greater than 0.4, then the *j*^th^ XP was associated to the *i*^th^ muscle group. Because EMG linear envelopes were normalized to the peak processed EMG values on a trial basis, it was expected that the amplitude of the final XPs did not affect the final muscle weighting distribution based on the employed 0.4 cut-off criterion. However, in order to assess this directly, an additional set of weightings was computed. In this, the weighting factors *W*_*j, i*_ (with 1 ≤ *j* ≤ 5, and 1 ≤ *i* ≤ 16) extracted from NNMF were first multiplied by the amplitude peak of the associated XP and then normalized as previously described in the Muscle Excitation Primitive Section. The two muscle weighting sets were then directly compared.

The *second test* assessed whether the MTU activations (i.e. resulting from the XP-to-activation mapping, Equations 2 and 3) predicted by the XP-driven model were similar to the MTU activations predicted by the EMG-driven model. Because the XP-driven model was calibrated on an individual to best match the variety of MTU and joint dynamics observed over all four calibration tasks (i.e., FW, RN, SS, and CO), it was expected that the MTU activations resulting from this task-generic XP-to-activation mapping well matched the EMG-driven MTU activations on average over the four motor tasks. However, because the XP-to-activation mapping was preserved across tasks, it was expected a lesser favorable matching with EMG-driven MTU activations across each individual trial.

The *third test* compared the joint moment prediction accuracy of the XP-driven and EMG-driven models. This assessed whether our proposed methodology could use a task-generic XP-to-activation mapping to predict task-specific joint moments simultaneously produced about the six considered DOFs.

The *fourth test* assessed whether the XP-driven musculoskeletal model was able to reproduce the similar variability observed in the joint moments predicted by the EMG-driven model as well as in the joint moments experimentally recorded. This question arises from the consideration that our proposed model is driven by the same set of XPs, which are only scaled in time to match the length of the trial-specific stance phase. A positive outcome of this test would give further confidence that the use of a subject-generic, task-generic, and low-dimensional XP set would not decrease the ability of the musculoskeletal model to produce movement-specific outputs and would not imply substantial loss of predictive ability with respect to using EMG recordings as an input to the model. Furthermore, this would imply that the predicted joint moment variability was the direct reflection of the predicted MTU kinematics which is the only model input that varies across trials as a function of the three-dimensional joint angles. Finally, this would support the hypothesis that dynamically different movements could emerge from the same locomotion program decoded in the spinal circuitries. For this purpose, we calculated and compared the standard deviation curves extracted from the joint moments predicted using the XPs and the EMG data as well as from those experimentally recorded.

In the *fifth test* the XP-driven musculoskeletal model calibration and execution time were examined. Calibration time was calculated as the time needed to calibrate the model on the eight calibration trials of each subject. Execution time was calculated as the average time needed to repeatedly compute one time point from all DOF joint moments 1000 times. Tests were performed on an 8 GB RAM Intel i7 CPU. If fast execution times were obtained from this test, it would imply the possibility of applying our proposed methodology for the on-line control of powered prostheses and orthoses.

## Results

**Figure A2:**
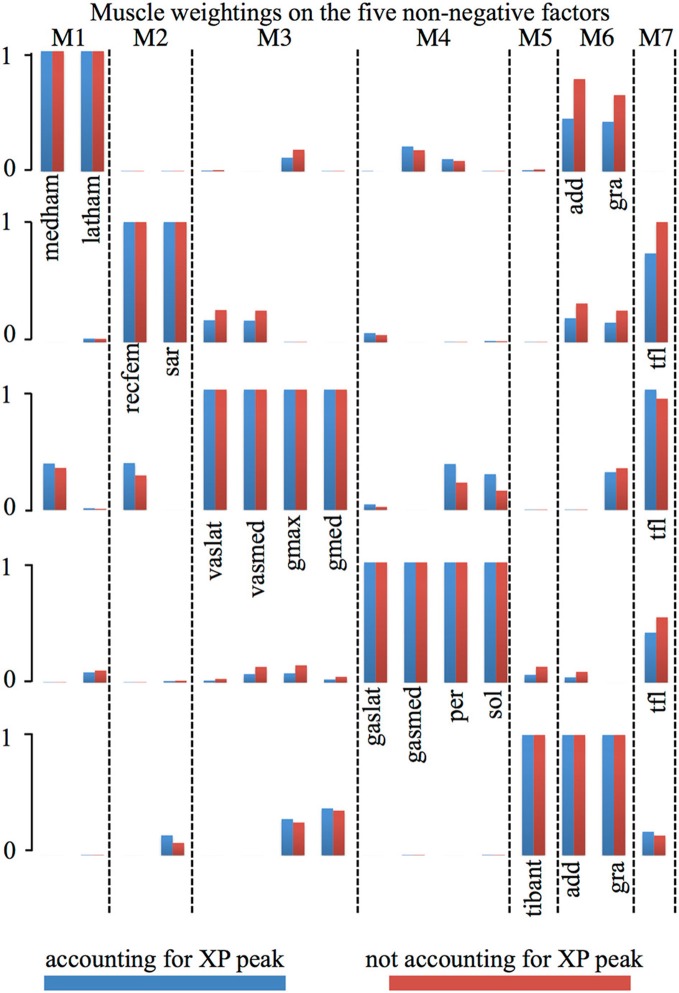
**Muscle weightings comparison**. Muscle weightings directly extracted from the non-negative matrix factorization algorithm (i.e., not accounting for XP peak, Figure 2A) are directly compared to those computed accounting for the excitation primitive (XP) peak amplitude (accounting for XP peak). Also see Table A1.

In the *first test* (Figures 2, A2, Tables 1, A1), the five experimentally extracted non-negative factors, and muscle group weightings accounted for the 89% of the experimental EMG data variability. Muscle groups were apportioned into seven modules according to the dominant weightings (Table 1). The NNMF (Figure 2A) revealed that the non-negative factor 1 was mostly responsible for the excitation of add (*W*_1_ = 0.77), medham (*W*_1_ = 1), latham (*W*_1_ = 0.64), and gra (*W*_1_ = 1). For the remaining muscle groups *W*_1_ ranged from 10^−5^ to 0.17. The non-negative factor 2 was mostly responsible for the excitation of recfem (*W*_2_ = 1), sar (*W*_2_ = 1), and tfl (*W*_2_ = 1). For the remaining muscle groups *W*_2_ ranged from 10^−5^ to 0.26. The non-negative factor 3 was mostly responsible for the excitation of gmax (*W*_3_ = 1), gmed (*W*_3_ = 1), tfl (*W*_3_ = 0.92), vaslat (*W*_3_ = 1), and vasmed (*W*_3_ = 1). For the remaining muscle groups *W*_3_ ranged from 10^−5^ to 0.33. The non-negative factor 4 was mostly responsible for the excitation of gaslat (*W*_4_ = 1), gasmed (*W*_4_ = 1), per (*W*_4_ = 1), sol (*W*_4_ = 1), and tfl (*W*_4_ = 0.55). For the remaining muscle groups *W*_4_ ranged from 10^−5^ to 0.13. The non-negative factor 5 was mostly responsible for the excitation of add (*W*_5_ = 1), gra (*W*_5_ = 1), and tibant (*W*_5_ = 1). For the remaining muscle groups *W*_5_ ranged from 10^−5^ to 0.3. The only muscle groups that received excitation from more than one XP were add, gra, and tfl. The alternative muscle weighting set, which we computed accounting for the XP peak amplitude (see Muscle Excitation Primitive Section), resulted in the same XP-to-MTU distribution that was obtained from the muscle weighting set that did not account for the XP peak amplitude. Figure A2 and Table A1 directly compare the values from the two muscle weighting sets. The parameterized XPs well fitted the experimental non-negative factors with *R*^2^ values ranging from 0.74 to 0.94, and *NRMSD* values ranging from 0.0003 to 0.25 (Figure 2B). Table 1 also summarizes how the five parameterized XPs were assigned to the 16 muscle groups and to the MTUs within and how these were apportioned into the seven muscle modules.

**Table 1:** **Muscle modules and their allocated excitation primitives (XP) to the 16 muscle groups and associated musculotendon units**.

**Muscle modules and excitation primitives (XP) used**	**Muscle groupings in the modules**	**Musculotendon units**
Module 1 using XP1:	medial hamstrings	biceps femoris short head (bfsh), biceps femoris long head (bflh)
	lateral hamstrings	semimembranosus (semimem), semitendinosus (semiten)
Module 2 using XP2:	rectus femoris	recfem
	sartorius	sar
	No experimental EMG	psoas
	No experimental EMG	illiacus
Module 3 using XP3:	gluteus maximus	gmax1, gmax2, gmax3
	gluteus medius	gmed1, gmed2, gmed3,
	No experimental EMG	gluteus minimus (gmin1, gmin2, gmin3)
	vastus lateralis	vaslat
	vastus medialis	vasmed
	No experimental EMG	vastus intermedius (vasint)
Module 4 using XP4:	gastrocnemius lateralis	gaslat
	gastrocnemius medialis	gasmed
	peroneus	peroneus longus (perlong), peroneus brevis (perbrev), peroneus tertis (perter)
	soleus	sol
Module 5 using XP5:	tibialis anterior	tibant
Module 6 using (XP1+XP5)/2:	hip adductors	adductor magnus (addmag1, addmag2, addmag3), adductor longus (addlong), adductor brevis (addbrev)
	gracilis	gra
Module 7 using (XP2+XP3+XP4)/3:	tensor fasciae latae	tfl

**Figure 3:**
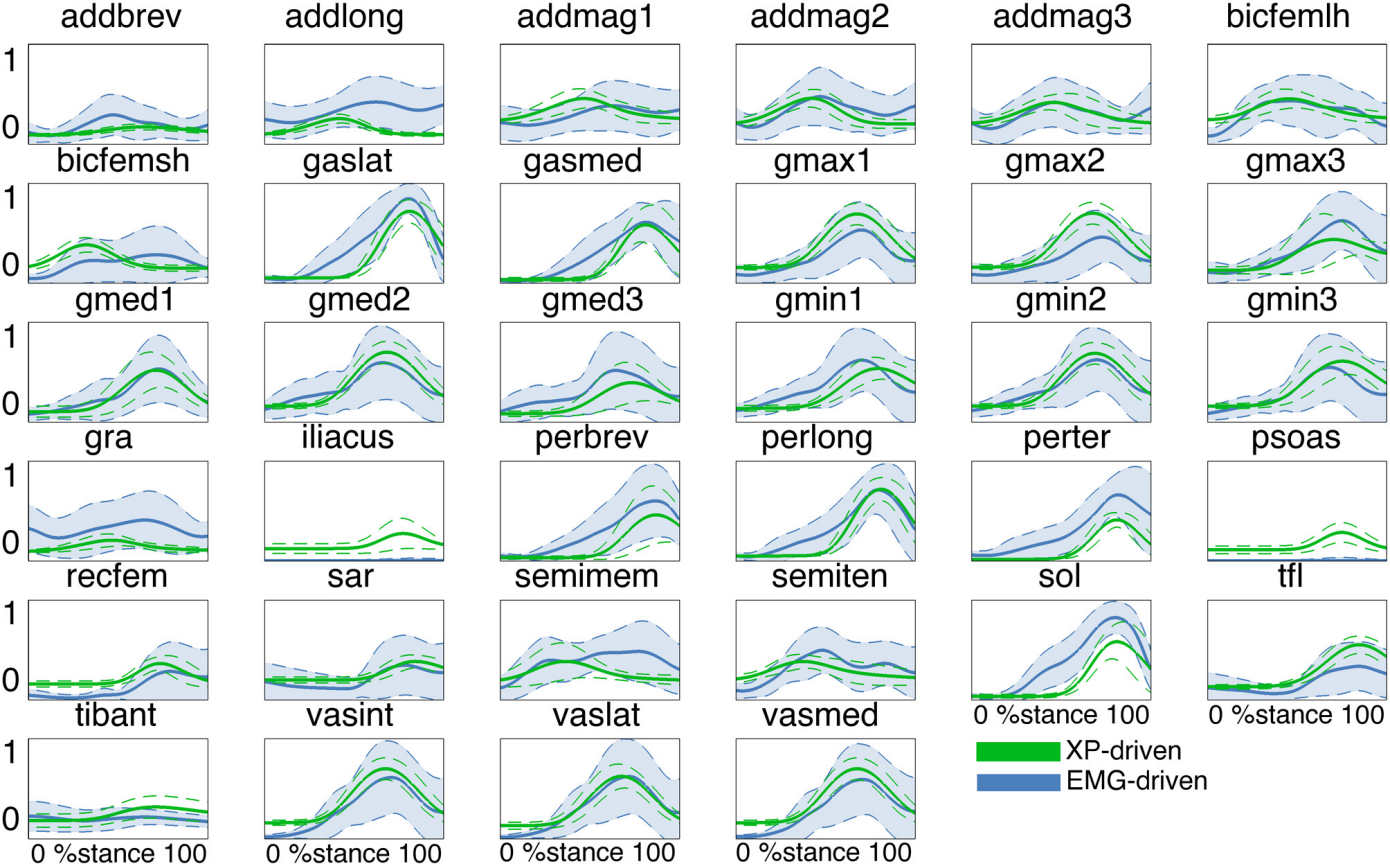
**Predicted musculotendon unit (MTU) activations**. The ensemble average (filled lines) and standard deviation (dotted lines) activation curves are depicted for the 34 MTUs included in the musculoskeletal model. Data are averaged across all trials and subjects. MTU names are defined as in Table 1. MTU activations are reported both from the estimates obtained from XP-driven and EMG-driven musculoskeletal models. The reported data are from the stance phase with 0% being heel-strike and 100% toe-off events.

**Figure 4:**
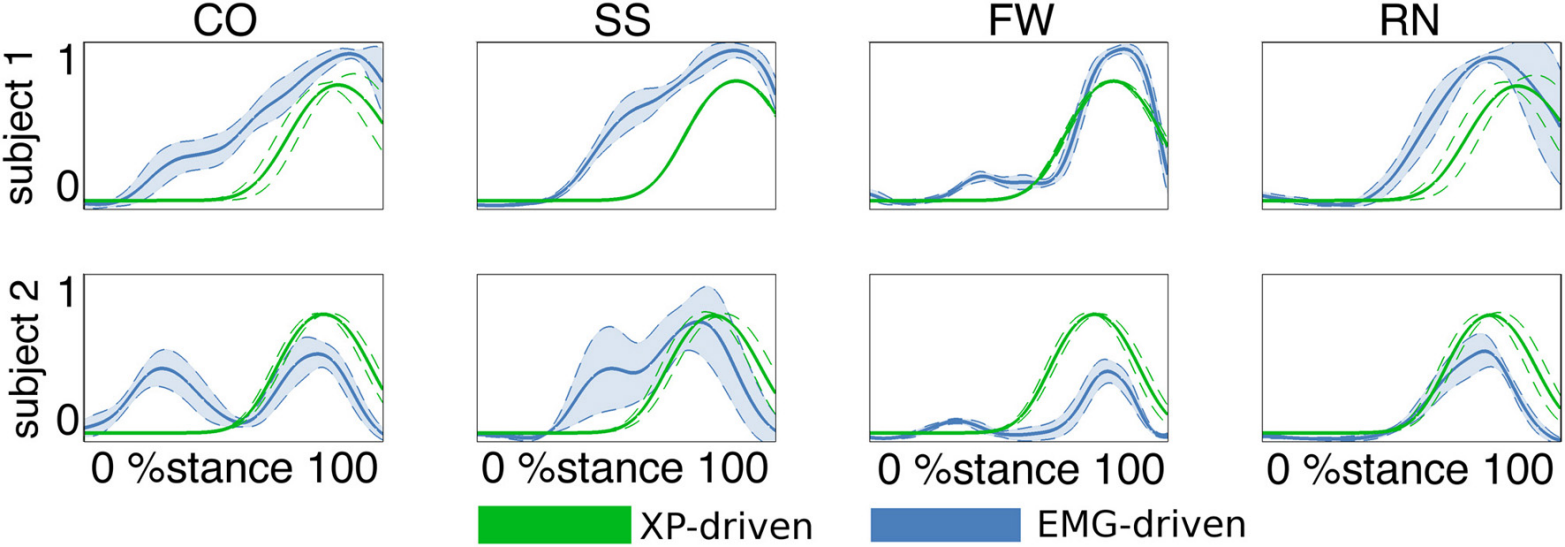
**Predicted musculotendon unit (MTU) activations**. The ensemble average (filled lines) and standard deviation (dotted lines) activation curves are depicted for the peroneus brevis MTU. Data are averaged across all trials within each motor task performed by the two subjects individually. Motor tasks included fast walking (FW), running (RN), crossover (CO), and sidestepping (SS) cutting maneuvers. Activations are reported both from the estimates obtained from XP-driven and EMG-driven musculoskeletal models. The reported data are from the stance phase with 0% being heel-strike and 100% toe-off events.

In the *second test* (Figures 3, 4, Table A2) the MTU-specific activations predicted by the XP-driven model closely matched the activations predicted using the EMG-driven model on average on all subjects and tasks (Figure 3). For this analysis the iliacus and psoas MTUs were not considered due to lack of experimental EMG data available. The *R*^2^ coefficient on the average MTU activations assumed values below 0.7 (i.e. between 0.07 and 0.67) for six MTUs only. For the remaining 26 MTUs, the *R*^2^ coefficient assumed higher values that ranged from 0.8 and 0.99. Similarly, the *NRMSD* coefficient on the average MTU activations assumed values above 0.3 (i.e. from 0.31 to 0.45) for five MTUs only. For the remaining 27 MTUs the *NRMSD* coefficient assumed smaller values that ranged between 0.057 and 0.25. Table A2 reports the detailed values from all MTUs for the *R*^2^ and *NRMSD* coefficients averaged across trials and subjects (i.e. results depicted in Figure 3). The proposed XP-driven model was also able to predict the activation of deeply located MTUs such as psoas and iliacus for which experimental EMG data were not available. In this, the ability of the XP-driven model to match, on average, the EMG-dependent MTU activation generated on the four motor tasks gives further confidence that the XP-dependent activations predicted for the deeply located MTUs may also be a reliable reflection of their average physiological behavior. However, further experimental validation is needed in this context. Figure 3 also shows that the XP-driven MTU activations assumed smaller variability (i.e. standard deviation range) with respect to the EMG-driven MTU activations. This is because of the use of a task-generic XP-to-activation mapping, which does not allow reproducing the whole range of the task-specific MTU activation patterns observed when using EMG data as input to the model. In this scenario, Figure 4 depicts the specific case of a representative MTU, i.e. the peroneus brevis. For this, *R*^2^ ranged from 0.58 to 0.98 while *NRMSD* ranged from 0.12 to 0.46 across subjects and tasks. Similar results were found for all remaining MTUs. Table A2 also reports the subject-specific *R*^2^ and *NRMSD* values for all MTUs averaged across all trials within each motor task and for each subject individually.

**Figure 5:**
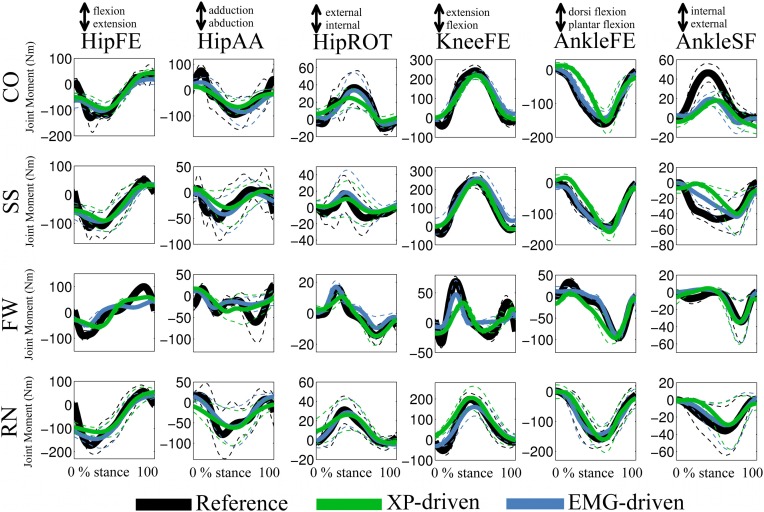
**Predicted and experimental joint moments**. The ensemble average (filled lines) and standard deviations (dotted lines) curves are depicted for the predicted (i.e. XP-driven and EMG-driven) and experimental (i.e., Reference) joint moments about six degrees of freedom (DOFs) including: hip flexion-extension (HipFE), hip adduction-abduction (HipAA), hip internal-external rotation (HipROT), knee flexion-extension (KneeFE), ankle plantar-dorsi flexion (AnkleFE), and ankle subtalar-flexion (AnkleSF). Results are shown for four motor tasks including: fast walking (FW), running (RN), side-stepping (SS), and cross-over (CO) cutting maneuvers. The reported data are from the stance phase with 0% being heel-strike and 100% toe-off events.

In the *third test* (Figure 5, Tables A3, A4), the XP-driven model predicted joint moments produced about the six lower extremity DOFs during the four motor tasks with comparable performance to the EMG-driven model (Figure 5). The *NRMSD* coefficient between predicted and experimental joint moments ranged from 0.048 and 0.46 when the EMG-driven model was used, whereas it ranged from 0.082 and 0.42 when the XP-driven model was employed. The *R*^2^ coefficient between predicted and experimental joint moments ranged from 0.2 to 0.99 when the EMG-driven model was used, while it ranged from 0.3 to 0.98 when the XP-driven model was used. Table A3 reports the full range of subject-specific and task-specific *R*^2^ and *NRMSD* values observed between the XP-driven model predictions and the experimental measurements. Table A4 reports the full range of subject-specific and task-specific *R*^2^ and *NRMSD* values between the EMG-driven model prediction and the experimental measurements. The weakest prediction accuracy was observed both in the XP-driven and EMG-driven models for the moments about HipAA during FW and to the moments about AnkleSF during CO and SS.

**Figure 6:**
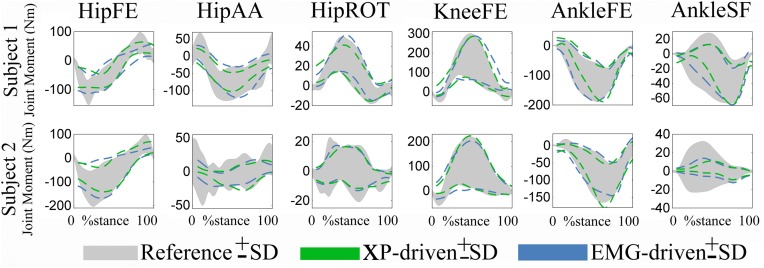
**Predicted and experimental standard deviations (SDs)**. Upper and lower SDs are reported subject-wise resulting from averaging across motor tasks. SDs are reported for the experimental joint moments (i.e., Reference, shaded area) about 6 degrees of freedom (DOFs) including: hip flexion-extension (HipFE), hip adduction-abduction (HipAA), hip internal-external rotation (HipROT), knee flexion-extension (KneeFE), ankle plantar-dorsi flexion (AnkleFE), and ankle subtalar-flexion (AnkleSF). The SDs are also shown for same joint moments predicted by the XP-driven and EMG-driven musculoskeletal models (i.e., dotted lines). The data are averaged across all trials and tasks. The data are reported from the two subjects over the stance phase, with 0% being heel-strike and 100% toe-off events.

In the *fourth test* (Figures 5, 6, Table 2), the upper and lower standard deviations (SDs) of the joint moments predicted using the XP-driven model assumed similar values to those predicted using the EMG-driven model and to those experimentally recorded. Figure 5 displays joint moment standard deviations task-wise, resulting from averaging across the subjects' performed trials within each specific task. Figure 6 and Table 2 report joint moment standard deviations subject-wise, resulting from averaging across all trials and tasks performed by each subject individually.

**Table 2:** **Coefficients of determination (*R*^2^) and the normalized root mean squared deviation (*NRMSD*) between the standard deviations *(SD)* of the joint moments measured experimentally and those predicted by the parameterized XP-driven model**.

	**Subject 1**				**Subject 2**			
	**Upper *SD***		**Lower *SD***		**Upper *SD***		**Lower *SD***	
	***R***^2^	**NRMSD**	***R***^2^	**NRMSD**	***R***^2^	**NRMSD**	***R***^2^	**NRMSD**
HipFE	0.93	0.11	0.88	0.15	0.89	0.13	0.91	0.16
HipAA	0.86	0.24	0.92	0.21	0.63	0.31	0.12	0.21
HipROT	0.93	0.15	0.96	0.12	0.89	0.18	0.75	0.19
KneeFE	0.98	0.08	0.95	0.13	0.99	0.06	0.76	0.19
AnkleFE	0.96	0.12	0.89	0.19	0.95	0.12	0.96	0.08
AnkleSF	0.95	0.24	0.69	0.31	0.73	0.36	0.13	0.48

The SD similarity observed between the XP-driven and the EMG-driven model estimates was within acceptable ranges. The *R*^2^ coefficients were always greater than 0.65 whereas the *RMSD* coefficients where always less than 0.26, about all DOFs, both across motor tasks (Figure 5) and subjects (Figure 6 and Table 2), and both for the upper and lower SDs. This gives further confidence that the use of our proposed XP-driven model can reproduce similar output variability both across subjects and tasks with respect to the use of experimental EMG recordings as an input to the musculoskeletal model.

Across tasks (Figure 5), the SD similarity observed between the XP-driven model estimates and the experimental data, had less favorable *R*^2^ and *RMSD* values that were observed about the AnkleSF during CO (i.e. *R*^2^ = 0.53 and *RMSD* = 0.4, upper SD) and SS (i.e. *R*^2^ = 0.01 and *RMSD* = 0.5, upper SD), and during FW about HipAA (i.e. *R*^2^ = 0.26 and *RMSD* = 0.36, lower SD), and KneeFE (i.e. *R*^2^ = 0.38 and *RMSD* = 0.22, upper SD). In the remaining cases, the *R*^2^ coefficients were always greater than 0.7 whereas the *RMSD* coefficients where always less than 0.21.

Across individuals (Figure 6 and Table 2), subject 1's *R*^2^ and *RMSD* coefficients were always greater than 0.69 and always less than 0.31, respectively. Less favorable values were obtained for subject 2 about AnkleSF (i.e. *R*^2^ = 0.13 and *RMSD* = 0.48, lower SD) and HipAA (i.e. *R*^2^ = 0.12 lower SD and *RMSD* = 0.31 upper SD). This may also explain the less favorable results from CO, SS, and FW about the same DOFs obtained when analyzing data task-wise (Figure 5). In the remaining cases, the *R*^2^ coefficients were always greater than 0.73 whereas the *RMSD* coefficients where always less than 0.19.

The *fifth test* revealed that the average calibration time for the XP-driven model was 21 h and 24 min. However, the calibrated open-loop models executed fast. The average open-loop execution time was 20.32 ± 0.2 ms.

## Discussion

Despite previous works used low-dimensional sets of impulsive curves to drive musculoskeletal models of the human lower extremity (Neptune et al., 2009; McGowan et al., 2010; Allen and Neptune, 2012), our proposed study combined muscle modularity with musculoskeletal modeling with the aim to address a number of novel questions. Our proposed study showed that one single low-dimensional set of single-impulse excitation primitives, or XPs, could be found to best fit the variety of muscle recruitment and excitation patterns observed from two subjects performing motor tasks biomechanically different from each other (i.e. FW, RN, SS, and CO). Once an XP set was defined, no further EMG recordings were needed for the model operation. The XP set determined the structure of a task-generic impulsive controller, which could be preserved across all tasks and subjects. The simplified structure of the task-generic impulsive controller was compensated by combination with movement-specific estimates of MTU kinematics. This allowed producing movement-specific estimates of MTU force and joint moment with no loss of accuracy with respect to those derived from experimental EMG data.

The application of the NNMF algorithm to the EMG data set showed that each XP excited one specific subset of muscle groups (Figures 2, A2, Tables 1, A1). The only groups that were excited by more than one XP were the hip adductors, the gracilis, and the tensor fasciae latae. This scheme of recruitment was determined based on a 0.4 cut-off criterion on the muscle weightings (see the Musculoskeletal Modeling Section) and was preserved across subjects and motor tasks, where the XPs were only scaled to match the stance phase length of each individual motor trial.

The XP-driven musculoskeletal model internal parameters were then calibrated to match the physiological characteristics of each subject recruited in the study (see Methods Section). This also allowed defining a finer non-linear mapping from the initial XPs to the 34 MTU-specific activations. The nature of this mapping represented a best fit of the variety of MTU excitation patterns observed during the four considered tasks and was specific to an individual. This was subsequently applied without further variations during the model validation step (i.e. model open-loop operation). It is worth stressing the fact that, in the context of muscle synergies, the XP-to-activation transformation (Equations 1, 2, and 3) reflects the weightings on the XPs (Figure 2A) because it allows for changes in the amplitude level and in the time shifting for a specific XP being refined on a specific MTU. One benefit of the XP-to-activation transformation is that it accounts for the dynamics of muscles (i.e. excitation, activation, and force) and joint (i.e. joint moment) as well as for the demand of the motor tasks being used for calibration. These factors are not accounted for by previously proposed dimensionality reduction methodologies that operate on EMG recording only. These include NNMF, principal component analysis, independent component analysis, or factor analysis.

During validation, the calibrated model was operated in open-loop on a set of novel trials that were not used during the calibration. However, the novel set of validation trials comprised the same motor tasks used for calibration (i.e. FW, RN, SS, and CO). In this process, the proposed model was driven by the five XPs and by the three-dimensional joint kinematics. In this, numerical optimization was not employed and experimental EMG data were not used as input.

Results demonstrated the proposed XP-to-activation transformation (Equations 1, 2, and 3) could properly solve for the neuromuscular redundancy by predicting a specific MTU activation solution (among the several possible ones) that well reflected a best fit of the different EMG-based MTU activation strategies observed during the four selected tasks (Figures 3, 4, Table A2). This result gains further importance if we consider that MTUs were driven by a set of Gaussian-shaped curves that were not linearly combined according to the muscle weightings (Figure 2 and Table 1). This proves that a subject-generic, task-generic, low-dimensional impulsive controller that recruits groups of MTUs with timing dependent on the stance phase can predict physiological MTU activation patterns that reflect subject-specific, task-specific EMG recordings of higher-dimensionality.

Results also showed that the proposed XP-driven model was able to predict joint moments that matched those experimentally measured from the six selected lower extremity DOFs with comparable accuracy to that associated to the EMG-driven model (Figure 5, Tables A3, A4). The ability of matching joint moments produced during different motor tasks implied that the proposed methodology was able to account for the different MTU activation strategies and contractile conditions associated to each motor task.

Furthermore, results showed that, although the excitation patterns driving the model (i.e. XPs) were the same across tasks (Figures 3, 4), the patterns of predicted joint moments varied across trials and this variability was in agreement with the variability observed both in the experimentally measured joint moments as well as in the joint moments predicted from EMG data (Figures 5, 6, Table 2). In this, the task-generic excitation patterns were continuously modulated by the movement-specific estimates of MTU kinematics (i.e. MTU length and moment arms, Figure 1A) derived from the experimental joint kinematics input (Sartori et al., 2012c). This modulation process took place both in the Musculotendon Dynamics component (Figure 1C) and in the Moment Computation component (Figure 1D). The Musculotendon Dynamics component combined MTU activation with MTU length to compute MTU force (Equation 4). The MTU force was then combined with the MTU moment arms to compute MTU moment. Therefore, the computation of MTU force and moment could be seen as a transformation of the task-generic MTU activation that accounted for movement-specific MTU kinematics, thus resulting in movement-specific estimates of joint moments (i.e. summation of MTU moments about a specific joint and DOF). This allowed compensating for the static behavior and for the simplified structure of the single XP-based controller.

Previous studies proposed analyses of muscles modularity using NNMF during locomotion tasks including walking (Ivanenko et al., 2005), running, and sidestepping cutting maneuvers (Oliveira et al., 2013). In these studies, five non-negative factors were identified and extracted. These reflected the recruitment of muscles in the lower and upper extremities. Our proposed study identified the same number of non-negative factors with respect to those in the literature (Ivanenko et al., 2005; Oliveira et al., 2013). However, our study only analyzed lower extremity muscles and solely during the stance phase. Furthermore, our extracted set of non-negative factors and weightings reflected the dynamics of four motor tasks simultaneously (i.e. FW, RN, SS, and CO) whereas the previously proposed studies (Ivanenko et al., 2005; Oliveira et al., 2013) analyzed a specific motor task individually, thus generating a task-specific set of non-negative factors and weightings. These differences were reflected in dissimilarities in the timing of the maximum peak amplitude observed across non-negative factors as well as in the distribution of weightings across muscles. Our extracted non-negative factors had maximum peaks localized from about 20% to 80% of the stance phase (Figure 2). The non-negative factors reported by (Ivanenko et al., 2005) during walking had the maximum peaks distributed from about 5% to the end of the stance phase. However, the non-negative factors 1 and 5 reflected the recruitment of trunk and arm muscles and their associated peaks were in the transition from the swing to the stance phase. Higher similarity in the maximum peak timing was observed in the inner non-negative factors including non-negative factor 2 (at about 45% and 55% in our study and in Ivanenko's et al. respectively), and non-negative factor 3 (at about 70% in both studies). A similar scenario was observed in Oliveira et al. (2013) during sidestepping, where non-negative factors had the maximum peaks distributed throughout the entire stance phase, with non-negative factors 1 and 5 reflecting the recruitment of the trunk muscles and implementing the transition across the stance and swing phase. In this, higher similarity in the maximum peak timing was observed in the inner non-negative factors that most reflected the recruitment of lower extremity muscles. These included non-negative factor 2 in our study and non-negative factor 3 in Oliveira et al. (2013) both at about 45% of stance. Furthermore, it also included non-negative factor 3 in our study and non-negative factor 4 in Oliveira et al. (2013) at about 70% and 65% respectively. The non-negative factors reported by Oliveira et al. (2013) during running had the maximum peaks distributed throughout the entire stance phase. In these, the recruitment of the trunk muscles was reflected by all five non-negative factors. The best similarity in the maximum peak timing was observed in the non-negative factors that most accounted for the recruitment of lower extremity muscles. These included non-negative factor 1 (at about 20% in both studies). Furthermore, it also included non-negative factor 3 in our study and non-negative factor 2 in Oliveira et al. (2013) at about 70 and 65% of stance respectively.

Future work is needed to further improve our proposed methodology. Experimental results showed that the trial-specific non-negative factors extracted from the different motor tasks had peaks that were substantially shifted in time depending on the nature of the task. This was especially evident in the third non-negative factor (Figure 2B). While the timing of the peaks for the trial-specific factors extracted from RN, SS, and CO was consistent, the timing of the peak for the trial-specific factors extracted from FW was anticipated by 30%. Future work is needed to allow adjusting the peak timing, magnitude, and bell width of the parameterized XPs during the model execution to implement the transition across tasks (i.e., RN, CO, and SS). Furthermore, the XP-to-activation transformation (Equations 1, 2 and 3) will be modulated across tasks thus allowing better representing the dynamics of individual movements. Moreover, the parameterized XPs could be, in the future, further modulated in time and amplitude based on biomechanical events triggering appropriate muscle reflexes to allow for adaptation to different gait dynamics and terrains.

The joint moment prediction accuracy tests (Figure 5 and Tables A3, A4) revealed that both the XP-driven and the EMG-driven models could not predict a substantial moment contribution about AnkleSF during the CO and SS cutting maneuvers tasks and about HipAA during FW. This may be explained by the fact that the MTUs currently included in the model, with AnkleSF and HipAA moment arms, accounted for the 80 and the 86% respectively of the total physiological cross sectional area. Future work should therefore include additional MTUs crossing the hip and ankle joints. The MTUs in the model with moment arms about the remaining four DOFs accounted for more than the 90% of the total physiological cross sectional area.

Our proposed methodology predicted joint moments during the stance phase only. The main reason for this was that calibration included trials of running, as well as sidestepping and crossover cutting maneuvers. For these motor tasks the swing phase occurred partially, or totally, out of the motion capture volume. Therefore there was an incomplete swing phase data available for calibration across trials. The second, although much lesser reason, was that joint moments were estimated using inverse dynamics, which strongly relies on the magnitude of GRFs (Delp et al., 2007). During the swing phase of locomotion, the GRFs are zero, which means the inverse dynamics calculations become highly sensitive to segmental inertial parameters that are difficult to measure *in vivo*. These include the segment mass, the location of the segment center of mass, and the mass moment of inertia (Lanovaz and Clayton, 2001; Delp et al., 2007), which were only scaled linearly to the subject's size (Delp et al., 2007). Inverse dynamics measurements of joint moments during the swing phase may therefore not be reliable and we preferred not to use these for the model calibration and for the subsequent validation step. Future work will focus on (1) using better methods for extracting subject-specific segmental parameters (i.e. using MRI), and (2) predicting joint kinematics, rather than joint moments, using full forward dynamics models (Barrett et al., 2007) or non-parametric methods such as Bayesian filtering (Ko and Fox, 2009). This will allow extending the analyses presented in this study to the whole gait cycle thus increasing the applicability of our proposed methodology.

The present results showed that our proposed methodology could predict MTU forces and joint moments within the range of DOFs, tasks, and gait cycle phases (i.e. stance phase) on which the model was calibrated. However, how the model extrapolates outside the range of these DOFs, tasks, and gait cycle phases is currently not know. Furthermore, it is not known whether or not new ranges of DOFs, tasks, and gait cycle phases would require updating the Gaussian curves in the impulsive controller accordingly. This requires an extensive and structured research, which was beyond the scope of this study. However, this will be important to be determined as the size of the calibration data set also affects the speed at which calibration can occur. Indeed, our proposed XP-driven model relies upon an off-line calibration procedure that is time consuming. On the other hand, the model execution was observed to be fast, i.e. in the order of 20ms per time frame. Future work should focus on the design of more efficient calibration algorithms. The use of MTU models that do not require an explicit integration of the MTU dynamics equations could considerably speed up the calibration process without loss of joint moment prediction accuracy as it was shown in Sartori et al. (2012b).

This work presented a study on two subjects only. Therefore, it may not be completely generalizable. However, the proposed XP-driven musculoskeletal model was scaled and then calibrated to the actual subjects to account for the subject-specific (1) anthropometry, (2) XP-to-activation mapping, and (3) MTU intrinsic properties. This allows our methods to be applied across individuals without relying on the existence of specific anthropomorphic models, while accounting for the individual's muscle activation patterns across multiple DOFs. This represents an improvement in current state of the art methodologies were the recruited subjects were chosen to be of similar build of the anatomical model (Lloyd and Besier, 2003; Martelli et al., 2011). However, a more general model validation across a larger number of individuals will be the subject of future work.

It is important noting that the aim of our proposed work was not that of addressing all limitations associated to excitation-driven modeling in one single study. Our aim was to demonstrate that a single impulsive controller could be used as input drive to large musculoskeletal models operating in open-loop across different motor tasks with no loss of accuracy with respect to using experimental EMGs. This supports the hypothesis that biomechanically different movements could emerge from the same locomotion program decoded in the spinal circuitries.

The proposed XP-driven model may have direct implications in the development of rehabilitation technologies. The proposed methodology could be, in the future, further extended to create generic XP sets descriptive of larger populations of subjects and motor tasks. Also, additional XP sets could be specifically created for different patient populations thus describing MTU recruitment patterns typically observed in different neurological or orthopedic conditions. This will give the potential possibility of extrapolating the generic XP-based impulsive controller to novel subjects (within a specific population) without needing to record further EMG data.

The ability of our proposed XP-driven model to predict physiological MTU activations and joint moments, will allow obtaining accurate predictions of the user's effort during dynamic movement. This will allow determining how muscles contribute to modulate joint compliance in locomotion (Rapoport et al., 2003; Cronin et al., 2011; Heitmann et al., 2011; Pfeifer et al., 2012). It will allow determining the heat released by muscles and the resulting metabolic energy consumption during movement (Sawicki and Ferris, 2009; Bisi et al., 2011; Krishnaswamy et al., 2011; Farris and Sawicki, 2012). Furthermore, it will allow determining the magnitude of reaction forces in the lower extremity joints (Winby et al., 2009; Lin et al., 2010; Fregly et al., 2012a; Modenese and Phillips, 2012; Manal and Buchanan, 2013). The ability of determining these variables will enable a number of applications in the field of neurorehabilitation technologies including (1) the design of powered prostheses that modulate joint compliance according to that modulated in the subject's contralateral leg, (2) the design of powered orthoses that can effectively reduce the energy consumption during locomotion, and (3) the monitoring and prevention of orthopedic conditions such as osteoporosis and osteoarthritis. In these scenarios, the proposed XP-driven model would only need direct recordings of joint angles and estimates of the gait cycle percentage as input. This would allow decreasing the input drive complexity and the number of needed sensors, thus increasing the robustness of the system with respect to the case requiring real measurements of EMG data.

The availability of the proposed methodology will facilitate the transition toward the design of human-inspired devices that can effectively embody the dynamics of the human neuromuscular control of movement without relying on explicit representations of task-specific control models.

### Conflict of interest statement

The authors declare that the research was conducted in the absence of any commercial or financial relationships that could be construed as a potential conflict of interest.

## Footnotes

^1^OpenSim release 2.2.0 available from: https://simtk.org/home/opensim

^2^Available from: https://simtk.org/home/nmblmodels/

^3^Code and documentation are freely available from: http://code.google.com/p/mcbs/

## List of acronyms and symbols sorted with respect to order of appearance in the text

MTU: Musculotendon units
EMG: Electromyography
XPs: Excitation primitives
DOFs: Degrees of freedom
FW: Fast walking
RN: Running
SS: Sidestepping cutting maneuvers
CO: Crossover cutting maneuvers
GRFs: Ground reaction forces
HipFE: Hip flexion-extension
HipAA: Hip adduction-abduction
HipROT: Hip internal-external rotation
KneeFE: Knee flexion-extension
AnkleFE: Ankle plantar-dorsi flexion
AnkleSF: Ankle subtalar flexion
add: Hip adductors
gmax: Gluteus maximus
gmed: Gluteus medius
gra: Gracilis
tfl: Tensor fasciae latae
latham: Lateral hamstrings
medham: Medial hamstrings
sar: Sartorius
recfem: Rectus femoris
vasmed: Vastus medialis
vaslat: Vastus lateralis
gasmed: Gastrocnemius medialis
gaslat: Gastrocnemius lateralis
per: Peroneus group
sol: Soleus
tibant: Tibialis anterior
IK: Inverse kinematics
ID: Inverse dynamics
RRA: Residual reduction analysis
NNMF: Non-negative matrix factorization
*m*: Number of muscles
*n*: The number of trial frames × number of trials × number of subjects
VAF: Variation accounted for
SSE: Sum of squared errors
TSS: Total sum of squares
*W*_*j,i*_: Muscle weighting
${\bar{W}}_{j,i}$: Normalized muscle weighting
*t*: Time
*h*: Gaussian curve peak height
*b*: Position of the center of the peak
*c*: Width of the curve bell
*s*: Vertical shift
*g(t)*: Fitted Gaussian curve
ℓ^*mt*^: Musculotendon unit length
*r*: Musculotendon unit moment arm
*x(t)*: EMG linear envelope
*u(t)*: EMG linear envelope processed by recursive filter
α, β_1_, β_2_: Recursive filter coefficients
*d*: Electromechanical delay
*a(t)*: Musculotendon unit activation
*A*: Shape factor
*f(v^m^)*: Fiber force-velocity relationship
*f_A_(l^m^)*: Fiber active force-length relationship
*f_P_(l^m^)*: Fiber passive force-length relationship
F^max^: Maximal isometric force
*f*(є): Tendon force-strain relationship
*v*^*m*^: Fiber contraction velocity
*l*^*m*^: Fiber length
*F*^*mt*^: Musculotendon unit force
*F*^*t*^: Tendon force
*F*^*m*^: Fiber force
ϕ*(t)*: Pennation angle
*M*_*X*_, with *X* = (HipFE, HipAA, HipROT, KneeFE, AnkleFE, AnkleSF): Predicted joint moment with respect to the degree of freedom *X*
*f*_*E*_: Calibration objective function
*E*_*X*_, with *X* = (HipFE, HipAA, HipROT, KneeFE, AnkleFE, AnkleSF): Error term with respect to the degree of freedom *X*
*l*^*t*^_*s*_: Tendon slack length
*l*^*m*^_*O*_: Optimal fiber length
*R*^2^: Square of the Pearson product moment correlation coefficient
*NRMSD*: Normalized root mean squared deviation
*N*: Number of frames in a specific curve
${\hat{M}}_{X}$, with *X* = (HipFE, HipAA, HipROT, KneeFE, AnkleFE, AnkleSF): Experimental joint moment with respect to the degree of freedom *X*
*g*^*j*^_exp_: Experimental Gaussian curve
*g*^*j*^_par_: Parameterized Gaussian curve
SD: Standard deviation

## Appendix

**Table A1:** **Comparison of muscle weightings normalized with and without accounting for the excitation primitive (XP) peak**.

	**Not accounting for XP peak**					**Accounting for XP peak**				
	***XP*1**	***XP*2**	***XP*3**	***XP*4**	***XP*5**	***XP*1**	***XP*2**	***XP*3**	***XP*4**	***XP*5**
medham	1.00	0.00	0.35	0.00	0.00	1.00	0.00	0.39	0.00	0.00
latham	1.00	0.03	0.01	0.10	0.00	1.00	0.03	0.01	0.08	0.00
recfem	0.00	1.00	0.29	0.00	0.00	0.00	1.00	0.39	0.00	0.00
sar	0.00	1.00	0.00	0.02	0.10	0.00	1.00	0.00	0.01	0.17
vaslat	0.01	0.27	1.00	0.03	0.00	0.01	0.18	1.00	0.02	0.00
vasmed	0.00	0.26	1.00	0.13	0.00	0.00	0.18	1.00	0.07	0.00
gmax	0.18	0.00	1.00	0.14	0.27	0.11	0.00	1.00	0.08	0.30
gmed	0.00	0.00	1.00	0.05	0.37	0.00	0.00	1.00	0.03	0.39
gaslat	0.00	0.06	0.02	1.00	0.00	0.00	0.08	0.05	1.00	0.00
gasmed	0.18	0.00	0.00	1.00	0.00	0.21	0.00	0.00	1.00	0.00
per	0.09	0.00	0.23	1.00	0.00	0.10	0.00	0.38	1.00	0.00
sol	0.00	0.01	0.16	1.00	0.00	0.00	0.01	0.30	1.00	0.00
tibant	0.02	0.00	0.00	0.13	1.00	0.01	0.00	0.00	0.06	1.00
add	0.77	0.32	0.00	0.09	1.00	0.44	0.20	0.00	0.04	1.00
gra	0.63	0.26	0.35	0.00	1.00	0.41	0.16	0.31	0.00	1.00
tfl	0.00	1.00	0.92	0.54	0.16	0.00	0.74	1.00	0.41	0.19

**Table A2:** **Coefficients of determination (*R*^2^) and the normalized root mean squared deviation (*NRMSD*) between the MTU activations predicted using the XP-driven and the EMG-driven models**.

	**Subject 1 (***R***^2^/*NRMSD*)**				**Subject 2 (***R***^2^/*NRMSD*)**				**Subjects1, 2**
	***CO***	***SS***	***FW***	***RN***	***CO***	***SS***	***FW***	***RN***	**All tasks**
addbrev	0.56/0.39	0.38/0.2	0.01/0.44	0.09/0.38	0.61/0.38	0.23/0.19	0.06/0.31	0.01/0.36	0.72/0.23
addlong	0.07/0.56	0.01/0.47	0.57/0.21	0.1/0.59	0.18/0.43	0.01/0.33	0.01/0.23	0.67/0.39	0.12/0.46
addmag1	0.16/0.38	0.06/0.34	0.48/0.36	0.02/0.38	0.77/0.2	0.39/0.42	0.05/0.59	0.01/0.31	0.42/0.2
addmag2	0.05/0.37	0.41/0.19	0.5/0.35	0.69/0.27	0.48/0.31	0.17/0.24	0.1/0.38	0.37/0.2	0.67/0.18
addmag3	0.41/0.24	0.79/0.32	0.19/0.52	0.69/0.17	0.35/0.39	0.01/0.29	0.1/0.26	0.49/0.33	0.83/0.12
bicfemlh	0.01/0.29	0.46/0.28	0.12/0.37	0.69/0.24	0.04/0.29	0.51/0.24	0.1/0.58	0.57/0.33	0.88/0.15
bicfemsh	0.33/0.4	0.22/0.46	0.07/0.5	0.36/0.5	0.31/0.3	0.05/0.36	0.9/0.33	0.05/0.46	0.03/0.32
gaslat	0.66/0.3	0.61/0.33	0.83/0.14	0.52/0.31	0.85/0.21	0.69/0.28	0.79/0.19	0.97/0.13	0.93/0.17
gasmed	0.77/0.23	0.46/0.37	0.58/0.26	0.9/0.18	0.78/0.22	0.82/0.21	0.92/0.1	0.99/0.17	0.94/0.17
gmax1	0.72/0.23	0.93/0.18	0.38/0.44	0.78/0.2	0.74/0.16	0.88/0.13	0.2/0.53	0.75/0.16	0.99/0.18
gmax2	0.54/0.31	0.76/0.28	0.37/0.47	0.98/0.22	0.74/0.16	0.88/0.12	0.19/0.52	0.76/0.16	0.97/0.23
gmax3	0.88/0.37	0.8/0.37	0.43/0.23	0.39/0.39	0.86/0.12	0.95/0.09	0.14/0.5	0.85/0.11	0.98/0.14
gmed1	0.92/0.28	0.97/0.25	0.01/0.44	0.96/0.14	0.85/0.15	0.66/0.23	0.01/0.44	0.93/0.14	0.98/0.06
gmed2	0.74/0.24	0.94/0.18	0.1/0.46	0.96/0.09	0.66/0.2	0.33/0.3	0.02/0.49	0.73/0.22	0.97/0.11
gmed3	0.77/0.37	0.93/0.33	0.01/0.43	0.96/0.21	0.1/0.37	0.08/0.51	0.16/0.55	0.1/0.35	0.93/0.22
gmin1	0.93/0.27	0.96/0.23	0.2/0.35	0.85/0.19	0.1/0.38	0.1/0.4	0.22/0.54	0.01/0.36	0.84/0.17
gmin2	0.87/0.21	0.97/0.15	0.18/0.39	0.93/0.1	0.76/0.15	0.65/0.23	0.04/0.52	0.78/0.17	0.98/0.09
gmin3	0.85/0.22	0.96/0.16	0.17/0.4	0.89/0.11	0.01/0.39	0.1/0.38	0.21/0.58	0.1/0.47	0.92/0.14
gra	0.04/0.42	0.12/0.4	0.51/0.4	0.1/0.37	0.43/0.63	0.31/0.62	0.02/0.35	0.79/0.57	0.58/0.44
perbrev	0.83/0.27	0.72/0.3	0.92/0.12	0.76/0.25	0.58/0.46	0.81/0.35	0.91/0.15	0.98/0.15	0.94/0.22
perlong	0.84/0.26	0.73/0.29	0.91/0.12	0.78/0.24	0.28/0.31	0.51/0.26	0.66/0.31	0.82/0.21	0.96/0.13
pertert	0.83/0.35	0.8/0.37	0.76/0.27	0.95/0.33	0.33/0.41	0.34/0.4	0.7/0.18	0.84/0.18	0.97/0.28
recfem	0.93/0.24	0.86/0.32	0.08/0.53	0.9/0.28	0.64/0.4	0.78/0.17	0.03/0.54	0.21/0.47	0.88/0.31
sar	0.34/0.29	0.7/0.36	0.02/0.59	0.61/0.33	0.07/0.31	0.9/0.1	0.22/0.48	0.27/0.34	0.93/0.15
semimem	0.28/0.43	0.1/0.29	0.17/0.33	0.2/0.46	0.01/0.43	0.11/0.48	0.04/0.28	0.5/0.51	0.33/0.31
semiten	0.02/0.41	0.82/0.19	0.5/0.28	0.23/0.35	0.78/0.21	0.76/0.24	0.86/0.35	0.56/0.29	0.67/0.2
sol	0.58/0.3	0.62/0.32	0.86/0.13	0.48/0.32	0.78/0.4	0.81/0.38	0.73/0.25	0.88/0.33	0.91/0.26
tfl	0.92/0.14	0.93/0.19	0.37/0.48	0.68/0.21	0.28/0.41	0.94/0.23	0.58/0.52	0.01/0.41	0.98/0.22
tibant	0.85/0.49	0.89/0.51	0.06/0.25	0.15/0.33	0.22/0.37	0.24/0.41	0.02/0.28	0.1/0.39	0.37/0.22
vasint	0.91/0.2	0.92/0.22	0.03/0.54	0.95/0.17	0.83/0.18	0.84/0.16	0/0.49	0.94/0.17	0.98/0.14
vaslat	0.9/0.25	0.92/0.28	0.04/0.5	0.97/0.14	0.91/0.17	0.91/0.16	0.01/0.5	0.89/0.19	0.98/0.09
vasmed	0.9/0.2	0.9/0.22	0.03/0.54	0.94/0.18	0.81/0.18	0.77/0.16	0.01/0.48	0.93/0.18	0.98/0.15

The R^2^ and the NRMSD are reported both for (1) the MTU activations averaged over all trials for a certain task and subject and for (2) the MTU activation values averaged over all trials and subjects.

**Table A3:** **Coefficients of determination (*R*^2^) and the normalized root mean squared deviation (NRMSD) between the joint moments measured experimentally and those predicted by the XP-driven model**.

	**Subject 1 (***R***^2^/*NRMSD*)**				**Subject 2 (***R***^2^/*NRMSD*)**			
	***CO***	***SS***	***FW***	***RN***	***CO***	***SS***	***FW***	***RN***
HipFE	0.79/0.17	0.84/0.13	0.8/0.15	0.75/0.17	0.83/0.12	0.87/0.13	0.76/0.16	0.82/0.17
HipAA	0.85/0.2	0.6/0.18	0.3/0.22	0.91/0.16	0.35/0.22	0.41/0.24	0.23/0.26	0.09/0.23
HipROT	0.78/0.23	0.91/0.13	0.86/0.12	0.79/0.18	0.56/0.17	0.73/0.2	0.74/0.15	0.81/0.15
KneeFE	0.96/0.12	0.94/0.1	0.39/0.23	0.91/0.18	0.95/0.08	0.92/0.1	0.62/0.19	0.84/0.17
AnkleFE	0.8/0.25	0.76/0.19	0.96/0.13	0.85/0.16	0.84/0.14	0.94/0.13	0.72/0.2	0.98/0.08
AnkleSF	0.9/0.29	0.36/0.33	0.82/0.14	0.66/0.25	0.35/0.42	0.3/0.57	0.8/0.17	0.91/0.25

Values are reported for joint moment estimates averaged over all trials within a certain task.

**Table A4:** **Coefficients of determination (*R*^2^) and the normalized root mean squared deviation (*NRMSD*) between the joint moments measured experimentally and those predicted by the EMG-driven model**.

	**Subject 1 (***R***^2^/*NRMSD*)**				**Subject 2 (***R***^2^/*NRMSD*)**			
	***CO***	***SS***	***FW***	***RN***	***CO***	***SS***	***FW***	***RN***
HipFE	0.85/0.15	0.81/0.15	0.64/0.19	0.76/0.18	0.8/0.14	0.87/0.13	0.73/0.21	0.86/0.13
HipAA	0.74/0.17	0.8/0.12	0.41/0.23	0.87/0.13	0.34/0.21	0.52/0.2	0.39/0.2	0.26/0.19
HipROT	0.97/0.07	0.89/0.19	0.9/0.15	0.93/0.09	0.53/0.19	0.77/0.18	0.87/0.14	0.88/0.12
KneeFE	0.9/0.15	0.86/0.15	0.64/0.17	0.94/0.11	0.96/0.1	0.93/0.13	0.84/0.18	0.93/0.14
AnkleFE	0.97/0.09	0.94/0.09	0.89/0.1	0.99/0.04	0.91/0.12	0.95/0.09	0.92/0.11	0.94/0.1
AnkleSF	0.82/0.31	0.78/0.16	0.91/0.12	0.93/0.11	0.88/0.34	0.2/0.46	0.78/0.18	0.68/0.26

Values are reported for joint moment estimates averaged over all trials within a certain task.
